# Uncertainty-Aware Semi-Supervised Method for Pectoral Muscle Segmentation

**DOI:** 10.3390/bioengineering12010036

**Published:** 2025-01-06

**Authors:** Yutao Tang, Yongze Guo, Huayu Wang, Ting Song, Yao Lu

**Affiliations:** 1 School of Computer Science and Engineering, Sun-Yat sen University, Guanghzou 510006, China; tangyt3@mail2.sysu.edu.cn (Y.T.); guoyz3@mail2.sysu.edu.cn (Y.G.); wanghy288@mail2.sysu.edu.cn (H.W.); 2 The Third Affiliated Hospital of Guangzhou Medical University, Guanghzou 510150, China

**Keywords:** deep learning, segmentation, uncertainty

## Abstract

The consistency regularization method is a widely used semi-supervised method that uses regularization terms constructed from unlabeled data to improve model performance. Poor-quality target predictions in regularization terms produce noisy gradient flows during training, resulting in a degradation in model performance. Recent semi-supervised methods usually filter out low-confidence target predictions to alleviate this problem, but also prevent the model from learning features from unlabeled data in low-confidence regions. Specifically, in medical imaging and other cross-domain scenarios, models are prone to producing large numbers of low-confidence predictions. To improve the quality of target predictions while utilizing unlabeled data more efficiently, we propose an uncertainty-aware semi-supervised method that incorporates the breast anatomical prior, for pectoral muscle segmentation. Our method has a typical teacher-student dual model structure, where uncertainty is used to distinguish between high- and low-confidence predictions in the teacher model output. A low-confidence prediction refinement module was designed to refine the low-confidence predictions by incorporating high-confidence predictions and a learned anatomical prior. The anatomical prior, as regularization of the target predictions, was learned from annotations and an auxiliary task. The final target predictions are a combination of high-confidence teacher predictions and refined low-confidence predictions. The proposed method was evaluated on a dataset containing 635 data points from three data centers. Compared with the baseline method, the proposed method showed an average improvement in DICE index of 1.76, an average reduction in IoU index of 3.21, and an average reduction in HD index of 5.48. The experimental results show that our method generalizes well to the test set and outperforms other methods in all evaluation metrics.

## 1. Introduction

Mammography is a procedure that uses low-energy X-rays to photograph the breast and is widely used for the early screening and diagnosis of breast cancer. The breast border, the nipple, and the pectoral muscle are three important anatomical markers on mammograms [1,2]. Specifically, the pectoral muscle is usually identified first in mammography-based screening and diagnosis. With the development of computer-aided detection technology, an increasing number of computer-aided detection systems have been applied in clinical practice, effectively reducing the burden of manual screening. Compared with the fatty tissue in the breast, the pectoral muscle usually has a higher intensity in mammograms, similar to that of breast masses and glandular tissue. This similarity may lead to false positives in automated scenarios, such as automatic breast mass detection and automatic breast density segmentation, where the pectoral muscle may be misidentified as the target. To avoid this, pectoral muscle removal is usually performed as a pre-task in computer-aided breast cancer detection and diagnosis algorithms [3,4].

Many automatic pectoral muscle segmentation methods have been proposed for mammography over the past few decades. Karssemeijer [5] used the Hough transform [6] to detect the pectoral boundary in breast parenchyma classification. Kwok et al. [1] used a cliff search method to improve the straight-line estimation obtained through the Hough transform and fitted it with polynomial curves. Ferrari et al. [7] designed a set of Gabor wavelet functions to enhance objects with a linear structure and then used the edge-flow method [8] to detect the pectoral boundary. Ma et al. [9] modeled the pectoral muscle segmentation problem separately using graph methods and fine-tuned the obtained pectoral muscle regions using an active contour model. In addition, many other methods based on thresholding [10,11,12,13,14,15], region-growing [16,17,18,19], clustering [20,21], graph methods [22], and boundary detection [23,24,25,26,27,28,29,30] have been proposed. Although these methods produce good results on most mammograms, they mostly rely on some simple assumptions about the intensity or shape of the pectoral muscle; thus, it is difficult to handle more complex cases. For example, threshold-based methods assume that the pectoral muscle has consistent and easily distinguishable high intensity; boundary detection methods assume that the boundary between the pectoral muscle and breast parenchyma has a cliff-like change in intensity within a small neighborhood; and, region-based methods, such as region growth, clustering, and graph methods, assume that the intensity in the pectoral muscle region is more consistent than that near its boundary. When mammograms are properly imaged, most of them can satisfy the above assumptions; however, in some mammograms, the pectoral muscle overlaps with its nearby lesions or glands, a situation in which the above assumptions cannot be validated. Incorrect breast compression and posturing during photography can also lead to changes in the intensity distribution of mammograms, in which case, the pectoral boundary is blurred or invisible [31]. In addition, most of the above methods assume that the pectoral boundary can be approximated as a straight line or simple curve, and they fit the pectoral boundary using straight-line estimation or polynomial curves. However, the shape of the pectoral muscle in mammograms is either convex, concave, or a combination of both, and polynomial curves cannot express this correctly.

In recent years, with the successful application of deep learning (DL) in semantic image segmentation, DL-based algorithms for pectoral muscle segmentation have been developed. Ma et al. [32] and Maghsoudi et al. [4] used U-Net [33] to segment the pectoral muscle. Rampun et al. [34] modified the holistically nested edge detection network [35] to detect the pectoral boundary. These DL-based methods are data-driven, and their optimization objectives are directly related to the task as loss functions. The convolutional neural networks (CNNs) used in these methods contain many learnable parameters and are automatically optimized during the training process, which avoids manual design features with oversimplified assumptions. These DL-based methods improve pectoral muscle segmentation results but still suffer from the following shortcomings:

1. The task of pectoral muscle segmentation can be split into two subtasks: detection of visible parts and estimation of invisible parts. The above methods only optimize for the final segmentation results, and the degree of optimization of the two tasks is difficult to balance during the training process. In most mammogram datasets, most images have a clear pectoral boundary, and the network tends to learn how to detect visible parts and ignore how to estimate blurred or missing parts. The performance of the model completely depends on the proportion of different types of data and the training tricks used, without addressing the problem through method design.
2. The weights of CNNs are highly correlated with the domain-relevant information of the training data, and the performance of the model may drop substantially when the training and test data are collected from different data centers.
3. Segmentation networks trained in a fully supervised manner require many pixel-level annotations. The datasets used by Ma et al. [32], Rampun et al. [34], and Maghsoudi et al. [4] contain 729, 1078, and 1100 labeled mammograms, respectively. In the field of medical image segmentation, the data annotations must be delineated by doctors specialized in the field, which is extremely costly in both time and labor.

In our previous work [36], a two-stage method for pectoral muscle segmentation was proposed to solve the first problem mentioned above using two sub-networks to solve the two subtasks separately. To solve the second and third problems, based on our previous work [36], this paper proposes a semi-supervised method for pectoral muscle segmentation in mammograms.

The key to the design of semi-supervised learning methods is the effective use of unlabeled data to improve the performance of the model. Many semi-supervised learning methods have been developed, among which the consistency regularity method is one of the most widely used. Consistency regularization is based on the manifold or smoothness assumption [37], which assumes that reasonable perturbations to the data should not cause changes in the model predictions. Accordingly, the consistency regularity term of the model can be constructed by constraining the output of the perturbed data.

Among the factors that affect the performance of the consistency regularity method, the degree of disagreement between predictions and the quality of predictions are two often-considered ones. Consistency regularization methods use the disagreement between predictions as the regularization term, and highly consistent predictions reduce the gain from the regularization term associated with unlabeled data. Meanwhile, if the prediction quality of unlabeled data is insufficient, the model suffers from consistency regularity and will struggle to learn new correct information, which is often referred to as confirmation bias [38]. To alleviate confirmation bias, many uncertainty-aware semi-supervised methods [39,40,41] simply filter out low-confidence predictions and train with only high-confidence predictions. This reduces not only the impact of confirmation bias but also the disagreement available for consistency regularization methods, and it does not make sufficient use of unlabeled data. Recent research on domain adaptation [42,43] has shown that models often produce low-confidence predictions when the data domain changes [44]. This implies that low-confidence prediction regions may contain domain-relevant information. Therefore, fully utilizing low-confidence predictions will effectively improve the generalization ability of the consistency regularization method on the test dataset.

The identification of blurred or missing boundaries is the most difficult part of pectoral boundary delineation and usually leads to low-confidence predictions in pectoral muscle segmentation. When doctors perform delineation, they consider not only the visible pectoral boundary and shape of the pectoral muscle but also the overall anatomical structure. Therefore, we propose a consistency regularization method that considers the uncertainty of pectoral muscle segmentation in mammograms. This method uses uncertainty to measure the confidence of the predictions and refines the low-confidence target predictions in unlabeled data so that the unlabeled data can be efficiently utilized. Our semi-supervised pectoral muscle segmentation method enables the model to learn domain-related information from the unlabeled data from the validation dataset during training, and improves the generalization ability of the model on the test dataset.

## 2. Materials and Methods

### 2.1. Datasets and Pre-Processing

The proposed uncertainty-aware semi-supervised method for pectoral muscle segmentation was evaluated using our in-house dataset. Mammograms were collected from three data centers using different mammography devices. The entire dataset contained 635 mammograms, with each data center providing approximately 200 mammograms. An experienced radiologist annotated the pectoral muscles of all mammograms in the dataset. Detailed information on the dataset is presented in Table 1.

To train the model effectively, all data in the dataset were pre-processed with orientation, size, and intensity normalization before training. First, all images in the dataset were resized to 448×320 by bilinear interpolation, which is a compromise between the hardware resources and image resolution, considering that the aspect ratios of the mammogram in all three data centers are close to 7:5, and adjusting to this ratio can reduce image distortion. Second, the intensity was normalized using a percentile normalization method, where the intensities below 10% and above 90% were set to the lowest and highest intensities, respectively, and then the overall intensity was mapped to [0, 1]. Finally, all mammograms were oriented according to the intensity distribution such that the pectoral muscle in all mammograms was located in the upper-left corner of the image. Informed consent was obtained from all subjects involved in the study. Written informed consent has been obtained from the patient(s) to publish this paper.

### 2.2. Overview of Methods

**Figure 1:**
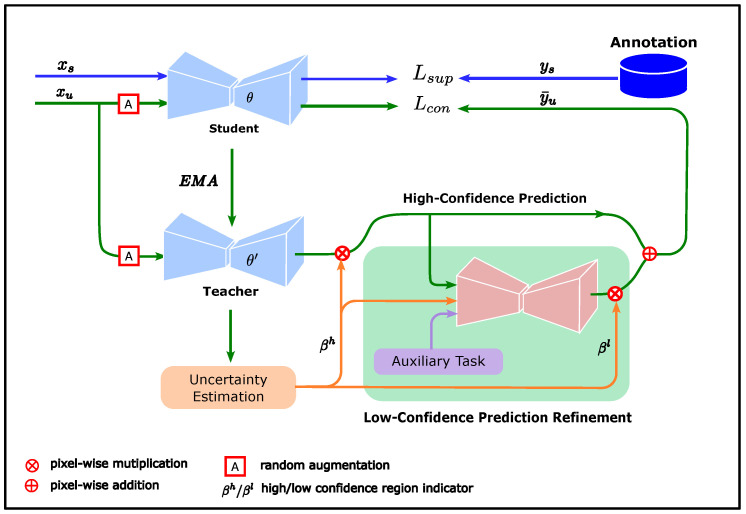
Overall framework of proposed method. The student model is trained by a supervised loss (blue lines) while being subjected to the consistency regularization constraint (green lines). The target predictions consist of high-confidence and low-confidence predictions. The high-confidence predictions come directly from the teacher predictions in the high-confidence region. The low-confidence predictions are refined by the low-confidence prediction refinement module, whose input consists of the high-confidence predictions, confidence region indicator, and breast segmentation result.

The proposed consistency regularization method considers uncertainty to measure the confidence of unlabeled data predictions and improve the contribution of unlabeled data in model training by refining low-confidence predictions using prior knowledge obtained from annotations. The overall framework of this method is shown in Figure 1.

The teacher and student models in Figure 1 were derived from the mean teacher (MT) method. The MT method is a consistency regularization method proposed by Tarvainen et al. [38], with a typical teacher–student dual model structure. The student updates the weights using the back-propagation algorithm, while the weights of the teacher are updated by the exponential moving average (EMA) of the student weights. Compared with the student, the teacher changes its weights more smoothly and is expected to provide more stable predictions. Therefore, the teacher predictions can be considered pseudo-labels for the corresponding unlabeled data to guide student training. The loss function $L_{MT}$ of the student model in the MT method comprises two components:

(1) $$L_{MT}=L_{sup-MT}+L_{con-MT},$$

where $L_{sup-MT}$ is the supervised loss from the labeled dataset, and $L_{con-MT}$ is the consistency regularization loss between the predictions of the unlabeled data and its pseudo-labels. $L_{sup-MT}$ and $L_{con-MT}$ are given in Equations (2) and (3), respectively.

(2) $$L_{sup-MT}=E_{x_{s}}\left(∥f\left(A\left(x_{s}\right),\theta \right)-y_{s}{∥}^{2}\right),$$

(3) $$L_{con-MT}=E_{x_{u}}\left(∥f\left(A\left(x_{u}\right),\theta \right)-y_{u}{∥}^{2}\right),$$

where $x_{s}$ and $x_{u}$ are the samples from the labeled and unlabeled datasets, respectively, $y_{s}$ is the label corresponding to $x_{s}$, and $y_{u}$ is the pseudo-label corresponding to $x_{u}$. f(·,θ) is the segmentation network with student model weight θ. A denotes a random perturbation of the input data. In the MT model, $y_{u}$ is obtained from the direct prediction of the teacher model:

(4) $$y_{u}=f\left(A\left(x_{u}\right),\theta^{\prime }\right),$$

where θ is the weight of the teacher network, updated by EMA.

Unlike the MT method, which uses the teacher predictions as pseudo-labels for student training, we want to obtain pseudo-labels that are more reliable than the teacher predictions to alleviate confirmation bias while making efficient use of the information from the unlabeled data. To achieve this, we must first identify which part of the teacher prediction $y_{u}$ is reliable and then improve the quality of the less reliable part of the predictions. Therefore, we use confidence to indicate whether a certain part of $y_{u}$ is reliable, where $\beta_{h}$ and $\beta_{l}$ denote the confidence indicators for the high- and low-confidence regions of $y_{u}$, respectively.

One way to measure confidence is uncertainty, which describes the degree of inconsistency in predictions and can be used as an estimation of confidence. High and low uncertainty can be considered predictions with low and high confidence, respectively. Accordingly, we define

(5) $$\beta_{h}=1\left(\Pi <\delta \right),$$

(6) $$\beta_{l}=1\left(\Pi \geq \delta \right),$$

where Π is the uncertainty of the teacher predictions, as output by the uncertainty estimation module in Figure 1, δ is a threshold, and 1 is the indicator function

(7) $$1\left(x\right)=\left\{\begin{array}{cc} 1, & \mathrm{if}\;x\geq 0,\\ 0, & \mathrm{otherwise}. \end{array}\right.$$

Once the high- and low-confidence regions of the teacher predictions $y_{u}$ are determined, the next step is to improve the quality of the low-confidence prediction. When performing pectoral muscle delineation, doctors first look for clearly visible pectoral boundaries in the mammogram images. Second, they consider factors such as the overall shape of the pectoral muscle and anatomical structure of the breast to determine the location of blurred or invisible boundaries.

Inspired by doctors’ delineation method, we designed a low-confidence prediction refinement module to learn how to refine low-confidence predictions using the visible boundaries, shape prior of the pectoral muscle, and structure prior of the breast. The visible boundary can be approximated using high-confidence teacher predictions as follows:

(8) $$y_{u}^{h}=y_{u}⊙\beta^{h},$$

where ⊙ denotes the Kronecker multiplier. The contrast between the breast and air is high in mammograms; therefore, a simple breast segmentation module was designed to obtain a rough contour of the breast, encoded as the feature $S_{breast}$; more details are given in Section 2.4. Regarding the shape prior of the pectoral muscle, many studies have assumed that the shape of the pectoral boundary can be approximated by a straight line or polynomial curve, but such a simplification may not match the real pectoral boundary shape. In our study, we used a GAN to learn the shape prior of the pectoral muscle.

GAN is a widely studied generative model that has been successfully used in many image-related fields, such as face synthesis [45] and style transfer [46]. GAN can learn a mapping from a source distribution to a target distribution without using pairs of data. We can use random noise as the input and annotations of the pectoral muscle as the target to train a GAN model; however, it is difficult to utilize the shape prior in this way. To make the shape prior easy to use, we use the high-confidence predictions and the breast shape as the input, with the annotation of the pectoral muscle as the target, and we train the GAN to refine the predictions. At this point, the shape prior of the pectoral muscle can be considered implicitly encoded in the GAN model. We also take the high-confidence region indicator $\beta_{h}$ as an input feature because, as a reasonable prediction, the output of the GAN model should be aligned with the teacher predictions in the high-confidence region. The low-confidence prediction refinement module in Figure 1 is the generator of the GAN, denoted as

(9) $$g=g(y_{p}^{h},\beta^{h},S_{breast}).$$

Once the output of *g* is obtained, the refined low-confidence predictions can be calculated by constraining the output to the low-confidence region

(10) $${\bar{y}}_{u}^{l}=g⊙\beta^{l},$$

while the target predictions are aligned with the teacher predictions in the high-confidence region

(11) $${\bar{y}}_{u}^{h}=y_{u}⊙\beta^{h}.$$

The final target predictions are simply the sum of Equations (10) and (11),

(12) $${\bar{y}}_{u}={\bar{y}}_{u}^{l}+{\bar{y}}_{u}^{h}.$$

Our loss function for student training is

(13) $$L_{Ours}=L_{sup-Ours}+L_{con-Ours},$$

(14) $$L_{sup-Ours}=E_{x_{s}}\left(∥f\left(A\left(x_{s}\right),\theta \right)-y_{s}{∥}^{2}\right),$$

(15) $$L_{con-Ours}=E_{x_{u}}\left(∥f\left(A\left(x_{u}\right),\theta \right)-{\bar{y}}_{u}{∥}^{2}\right).$$

### 2.3. Uncertainty Estimation

An important part of our method is the estimation of uncertainty, which is used to measure the confidence of the teacher predictions. Currently, many uncertainty estimation methods applied in image segmentation and image detection [39,47] are based on Monte Carlo dropout [48]. When dropout is applied, a certain percentage of the weights in the specified layer is randomly set to zero, which can be regarded as a sampling process of the model weights. Thus, a batch of different predictions with the same input can be obtained for uncertainty estimation. However, dropout is usually applied to only a few specific layers of the model, and for the layers without dropout applied, the model weights always remain constant, which may lead to over-confident predictions in uncertainty estimation.

To address this problem, instead of sampling weights from a single model, we save the model weights at different moments in the optimization process. Specifically, after the validation set loss is stabilized during training, the model weights are saved at fixed epochs, denoted as $\left\{\theta_{i}^{\prime }\right\}$. The batch of predictions used for uncertainty estimation can be obtained using the model with different weights as in

(16) $$y_{u}^{i}=f\left(x_{u},\theta_{i}^{\prime }\right).$$

After obtaining a batch of predictions for each unlabeled data point, the uncertainty can be estimated in various ways, such as through variance [36] or entropy [39,40]. Entropy measures the degree of information confusion, and higher entropy represents a prediction with higher uncertainty. For our method, we use entropy to estimate uncertainty:

(17) $$\Pi_{pixel}=-\sum_{i=1}^{K}p_{i}\log p_{i},$$

where $p=\mathrm{Softmax}(y_{u}^{i})$, and *K* is the number of models giving predictions.

The uncertainty estimation in Equation (17) uses a pixel-independent method that does not consider the pectoral muscle shape. If Equation (17) is applied directly, the obtained confidence region will be highly scattered. When performing manual delineation, the clarity of the pectoral boundary is usually the dominant factor in determining whether the delineation is reliable, while pixels away from the boundary are usually ignored. It is easy to see that there are some semantic discrepancies between the uncertainty estimated in a pixel-independent manner and the basis for judging whether the manual delineation is reliable. To eliminate this discrepancy, we propose row-based uncertainty to represent the confidence of the pectoral muscle predictions.

Row-based uncertainty is a simplification of the uncertainty calculation using contour coordinates, which is based on the shape of the pectoral muscle in a mammogram. Assuming that all mammograms are properly oriented, there should be at most one intersection point with the pectoral boundary in each row of the mammogram because the pectoral muscle does not curl excessively during mammography. Given that the confidence of annotations is usually related to the clarity of the pectoral boundary, this can be approximated by the width of the high-response regions in the pixel-independent uncertainty near the boundary. Once the row-based uncertainty, denoted as Π, is obtained, the high-confidence and low-confidence regions can be determined using Equation (5) and Equation (6), respectively.

Following the above design guidelines, the calculation of the row-based uncertainty Π first requires the width of the high-response region near the boundary in $\Pi_{pixel}$ to be obtained. There are three problems to solve here: locating the boundary, obtaining the high-response region, and computing the width of the high-response region. We observe that the clearly visible boundary has a smaller width in the high-response region with pixel-independent uncertainty $\Pi_{pixel}$. Based on this observation, we simplify the algorithm by directly determining the possible locations of the clear boundary; the rows where the clear boundaries are located belong to the high-confidence region, and the remaining rows belong to the low-confidence region.

**Figure 2:**
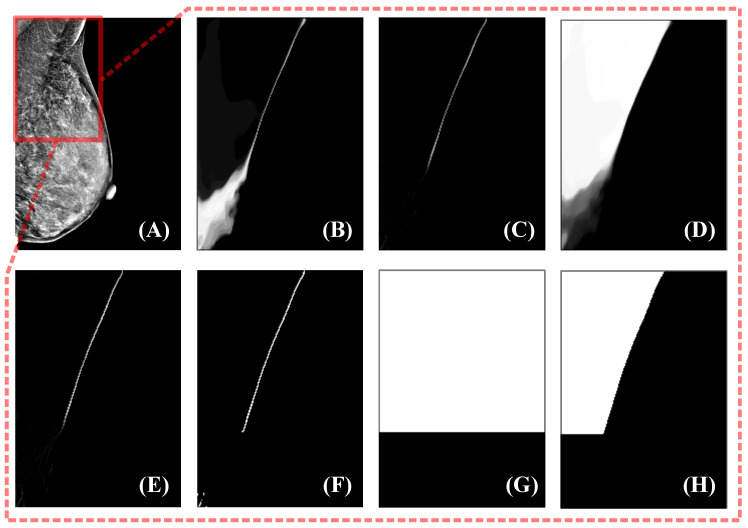
Uncertainty estimation method. The mammogram to be segmented is shown in (**A**), and the fields of view in (**B**–**H**) correspond to the red box in (**A**). (**B**) is the pixel-independent uncertainty response obtained by the uncertainty estimation module. Using the top hat to remove the structures with larger widths yields (**C**). Next, the CESF method is applied to (**C**) to obtain an enhanced response (**E**). Then, thresholding and maximum connected region acquisition are used to find a binarized boundary, as in (**F**). (**G**) is the high-confidence region indicator, and the high-confidence teacher prediction is obtained by multiplication of (**G**) and the teacher prediction (**D**).

The entire algorithm can be described as follows: First, the response of $\Pi_{pixel}$ is processed by the top hat morphological operation, where a larger response can be obtained for structures with smaller widths. Next, the response of the top hat is processed using thresholding to obtain smaller-width structures and suppress noise. At this point, the location of the clear boundary is clearly accessible; however, there is still noise and other tissue structures similar to the pectoral muscle, such as glandular tissue near the pectoral muscle, which may also have a high response. To eliminate these disturbances as much as possible, we choose the largest connected region as the pectoral boundary selected by the algorithm. Experiments show that such a selection strategy can meet our requirements and is sufficiently robust. Before thresholding the top hat responses, we process the acquired response maps using coherence-enhancing shock filters (CESF) [49]. This is because of the random nature of model selection in uncertainty estimation, which can lead to the random presence of low-response pixels in high-response areas. If processing is not performed, the robustness of the large connected region selection strategy is reduced. CESF is an anisotropic diffusion method proposed by Weickert [49] that determines the direction of diffusion based on the gradient of the image within a larger neighborhood. This method is commonly used for image processing with flow-like structures, such as fingerprint recovery, and is therefore also applicable to the processing of pectoralis major boundaries. The experimental results for this algorithm on sample data are shown in Figure 2.

### 2.4. Low-Confidence Prediction Refinement

To refine the low-confidence predictions, we use GAN to learn the shape prior of the pectoral muscle. GAN is a generative model proposed by Goodfellow [50] that contains two components: a generator and a discriminator. The generator learns how to generate target samples, while the discriminator determines whether the input samples are from the generator or the target dataset, and the two models are trained using a min-max game. At the end of the training, the data generated by the generator can follow the real data distribution, making the discriminator unable to determine the source of the input samples.

In our method, the generator of the GAN is used to implement the low-confidence prediction refinement module *g* in Equation (9), and the output of *g* is made to satisfy the shape prior of the pectoral muscle by training the GAN. The input of the low-confidence prediction refinement module *g* has three features: high-confidence teacher predictions $y_{u}^{h}$, high-confidence region indicator $\beta^{h}$, and breast shape feature $S_{breast}$. The acquisition of $y_{u}^{h}$ and $\beta^{h}$ was described in the previous subsections, and this subsection describes how to obtain $S_{breast}$.

In mammograms, there is a large intensity difference between the breast and air. After pre-processing by intensity normalization and label removal, the breast can be obtained through a sequence of morphological operations, including global thresholding, hole filling, and acquisition of the maximum connected area. Next, we must consider how to encode the segmentation result of the breast into a good feature for pectoral muscle prediction refinement. Intuitively, using the distance from the pixel to the breast boundary is a reasonable way to encode the shape of the breast, as the breast boundary and pectoral boundary are important anatomical benchmarks for localization in breast cancer diagnosis. However, factors such as the size of the breast and selection of the field of view at the time of photography can have a large impact on the distance function. In our experiments, we found that a few mammograms showed large variations in the distance function response owing to inappropriate shooting poses, such as including too much of the axilla, which interfered with the results. Therefore, we directly used the breast segmentation result as the encoding of breast shape $S_{breast}$ and trained the network to automatically learn the relationship between the pectoral boundary and breast.

GAN suffers from pattern collapse during training [51]. To solve this problem, the Wasserstein GAN (W-GAN) improves GAN by constraining the discriminator gradient amplitude in the loss function [51,52], thereby stabilizing the training process. In our method, we use the training method of W-GAN to train our model. The loss functions of W-GAN are

(18) $$L_{D-WGAN}=\underset{L_{D-Orig}}{\underset{︸}{-E_{\tilde{y}∼P_{G}}\left[D\left(\tilde{y}\right)\right]-E_{y∼P_{r}}\left[D\left(y\right)\right]}}+\underset{L_{D-Grad}}{\underset{︸}{\lambda E_{\hat{y}∼P_{\tilde{y}}}\left[{\left({∥\nabla_{\hat{y}}D\left(\hat{y}\right)∥}_{2}-1\right)}^{2}\right]}},$$

(19) $$L_{G-WGAN}=\underset{L_{G-Orig}}{\underset{︸}{-E_{\tilde{y}∼P_{G}}\left[D\left(\tilde{y}\right)\right]}},$$

where $\tilde{y}=G\left(x\right)$, *x* denotes the input to the generator, $\hat{y}=\alpha \tilde{y}+\left(1-\alpha \right)y$, and $P_{G},P_{r},P_{\hat{y}}$ denote the distributions of G(x), true data, and $\tilde{y}$, respectively. λ is the weight parameter for the gradient penalty term. $L_{D-Orig},LG-Orig,L_{D-Grad}$ are the generator loss and discriminator loss of the original GAN [50] and gradient penalty term of the W-GAN, respectively.

To constrain the output of *g* to be aligned with the teacher predictions over the high-confidence region, we add an additional constraint term to the generator loss of W-GAN, and the discriminator loss is kept consistent with W-GAN. The discriminator and generator losses for training *g* are

(20) $$L_{D-WGAN}=\underset{L_{D-Orig}}{\underset{︸}{-E_{\tilde{y}∼P_{G}}\left[D\left(\tilde{y}\right)\right]-E_{y∼P_{r}}\left[D\left(y\right)\right]}}+\underset{L_{D-Grad}}{\underset{︸}{\lambda E_{\hat{y}∼P_{\tilde{y}}}\left[{\left({∥\nabla_{\hat{y}}D\left(\hat{y}\right)∥}_{2}-1\right)}^{2}\right]}},$$

(21) $$L_{G-WGAN}=\underset{L_{G-Orig}}{\underset{︸}{-E_{\tilde{y}∼P_{G}}\left[D\left(\tilde{y}\right)\right]}}+\underset{L_{G-Con}}{\underset{︸}{{∥\tilde{y}⊙\beta^{h}-y_{u}^{h}∥}_{2}^{2}}},$$

where $L_{G-Con}$ is the consistency loss over the high-confidence region.

**Figure 3:**
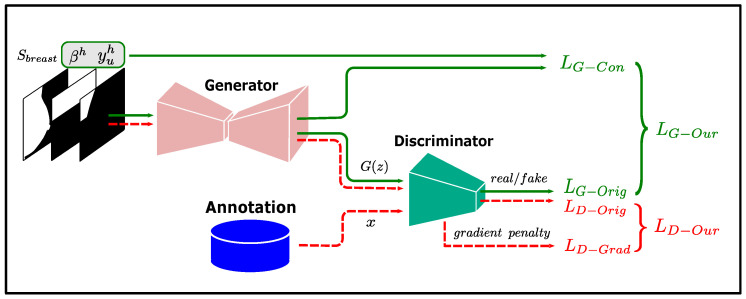
Training flow of the low-confidence prediction refinement module.

Pectoral boundaries in most mammograms in our dataset are clearly visible, and training directly with high-confidence teacher predictions would make it difficult for the network to learn how to properly handle the inputs with a lower percentage of high-confidence predictions. Therefore, we randomly erase a certain percentage of the pectoral muscle annotations as input features instead of $y_{u}^{h}$. The training flow of the low-confidence prediction refinement module is shown in Figure 3.

### 2.5. Data Analysis

To evaluate the performance of the model, we used three metrics: Dice coefficient(Dice), Intersection over Union (IoU), and Hausdorff distance (HD). The Dice coefficient measures the area overlap between the segmentation result and the true value. The IoU measures the similarity between the predicted and ground truth regions. HD assesses the maximum surface distance, in particular, the maximum distance from one point in one set to the nearest point in the other set.

Dice is the most frequently used metric in image segmentation tasks, and it is a set similarity metric that is usually used to calculate the similarity of two samples.

(22) $$Dice=\frac{2|A\cap B|}{A\cup B}$$

where A is the set of predicted values, B is the set of true values, and the numerator is the intersection between A and B.

The IoU score is a standard performance measure for object category segmentation problems. Given a set of images, the IoU measurement gives the similarity between the predicted and ground truth regions of objects present in the set of images and is defined by the following equation:

(23) $$IoU=\frac{TP}{FP+TP+FN}$$

where TP, FP, and FN represent true positive, false positive, and false negative counts, respectively.

The Hausdorff distance is a measure to describe the degree of similarity between two sets of points. It is a definition of the distance between two sets of points. Suppose there are two sets of sets A and B, then the Hausdorff distance between these two sets of points is defined as:

(24) HD(A,B)=max(h(A,B),h(B,A))

(25) $$h\left(A,B\right)=max_{a\in A}\left\{min_{b\in B}∥a-b∥\right\}$$

(26) $$h\left(B,A\right)=max_{b\in B}\left\{min_{a\in A}∥b-a∥\right\}$$

### 2.6. Implementation Details

The method was implemented using PyTorch, and all models were trained using the ADAM [53] optimizer. Before the images were fed into the networks, random augmentations were applied to the images, including random contrast adjustment and the CLAHE method. During training, the student and teacher models save their weights every five epochs, and finally, the optimal weights of the models are selected according to the validation loss.

## 3. Results

### 3.1. Quantitative Evaluation

Our method uses three-fold cross-validation, with the data from each data center serving as one fold. Only data from a single data center were used for each training, and data from the other two data centers were used as the validation and test sets, respectively. The experimental settings used in this study are described in Table 2.

We compared our method with the fully supervised and semi-supervised MT methods. In addition to DICE, IoU, and HD metrics, we adopted the overall unacceptable segmentation ratio (OUSR) to measure the performance of the three methods. “Unacceptable” segmentation was defined as a segmentation result with a DICE value less than 85% and as unacceptable as evaluated by a senior physician. The underlying segmentation networks for the three methods were all U-Net with the same architecture. A comparison of these results is presented in Table 3.

The experimental results show that our method generalizes well to the test set and outperforms other methods in all evaluation metrics. As shown in Table 3, the mean DICE exceeded 0.94 for all three groups of experiments for the fully supervised method. This suggests that a higher proportion of mammograms in our randomly sampled dataset had pectoral muscles that were easily identified, which is consistent with experience in clinical practice. These mammograms are usually taken in strict accordance with mammography standards so that a clear view of the entire pectoral boundary can be seen. However, the fully supervised method in Table 3 achieved an OUSR of 55/635≈8%, which indicates that there is still a certain percentage of mammograms in the dataset with unsatisfactory segmentation results. Possible reasons for this situation are insufficient training data, low percentage of mammograms with blurred pectoral boundaries, and degraded model performance caused by switching the data center of the test set.

The MT method additionally uses the data of the validation set for semi-supervised training, which provides additional constraints for the model through the consistency regularization term and alleviates the problem of insufficient data volume. At the same time, the model can obtain domain-related information from the validation set data, which increases the generalization ability of the model on the test set. Table 3 shows that the MT method reduces the overall number of unacceptable segmentations compared with the fully supervised method. However, the MT method is affected by the quality of the prediction of unlabeled data, which is greatly affected by the confirmation bias. Specifically, the MT method was better than the fully supervised method in individual indicators, but overall, the performance of the MT method in the three groups of experiments was not significantly different from that of the fully supervised method.

Our uncertainty-aware semi-supervised method is a consistency regularization method based on the MT method; therefore, it inherits the advantages of the MT method. Meanwhile, our method uses uncertainty to measure the confidence of the predictions and refines the low-confidence predictions using learned prior knowledge, thereby reducing the impact of confirmation bias. Table 3 shows that the proposed method achieved optimal results in all three groups of experiments. It can be seen that the method proposed in this study effectively improves the model generalization performance. In terms of OUSR, our method reduces this metric by more than 50% compared to fully supervised methods. It can be seen that our method performs well in improving the low-confidence prediction results.

### 3.2. Qualitative Evaluation

**Figure 4:**
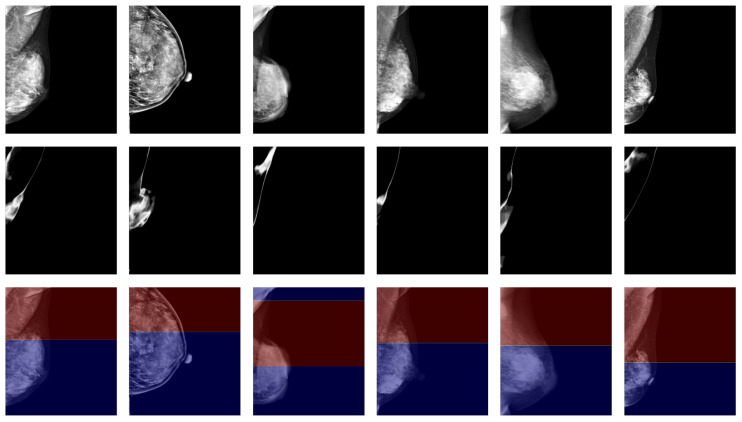
Confidence region as an estimation of the degree of clarity. The images in the first row are target mammograms. The images in the second row are the pixel-independent uncertainty responses. The red and blue regions in the third row represent the high-confidence and low-confidence regions, respectively.

When performing pectoral muscle delineation, the doctor first delineates the part of the pectoral muscle with clearly visible boundaries and then estimates the parts that are difficult to identify in the mammogram by incorporating a priori knowledge of the anatomical structure. The degree of clarity originates from the intuition of human vision, and to draw on the doctor’s experience, we used high- and low-confidence regions as the estimation of clearly visible and blurred regions, and we calculated the high- and low-confidence regions using uncertainty. In our experiments, we found that such an estimation can match intuitive judgments. Figure 4 shows the results for some samples in the dataset using confidence regions as an estimation of the degree of clarity.

## 4. Discussion

Deep learning methods typically assume that the data in the training and test sets follow the same distribution; however, for applications related to medical imaging, this cannot be assumed. One reason for the variation in the data distribution for medical imaging is that imaging devices in different data centers often have different built-in photography parameters and processing algorithms. In our experiments, we collected data from different imaging devices at three data centers to simulate real-world scenarios. The training and test sets used in each of the three groups of experiments are from different data centers, which requires our model to have good generalization ability to the test set. Our proposed method improves the generalization ability of the model on the test set in the following ways:

1. Consistency regularization constraints are constructed using unlabeled data from the validation set to participate in model training. In deep learning models, it is often assumed that the training set and the dataset have the same data distribution, an assumption that is often difficult to satisfy in practice, especially because medical imaging data often contain information related to the data domain introduced by specific device parameters. If the loss function contains only information relevant to the training set, the performance of the model on the test set largely depends on its similarity to the training set data. By adding a consistency regularization term constructed from the validation set data to the loss function, the output of the model on different datasets is constrained by the consistency regularization. The results in Table 3 show that both the MT method and our proposed method reduce the OUSR in the test set in this way.
2. Leveraging domain-related information contained in the low-confidence prediction region. Recent studies on domain adaptation [42,43] show that models tend to produce low-confidence predictions when the target and source data domains differ. This suggests that low-confidence predictions may be caused by the presence of domain-related information in the target data domain that has not been learned by the model. Directly constructing consistency regularization terms using these low-confidence predictions can cause the model to suffer from confirmation bias. Meanwhile, using the strategy of discarding low-confidence predictions, as in recent studies, loses the opportunity to fully learn the domain-relevant information from the dataset. We draw on the experience of doctors when performing delineation and refine the low-confidence predictions using high-confidence predictions as well as the learned prior to enable the model to fully learn the domain-relevant information from the dataset. In the experiments, our method outperformed the other two methods in all evaluation metrics.
3. The utilization of prior knowledge reduces the dependence of target predictions on the teacher predictions and improves the accuracy and stability of model training. The GAN used in the low-confidence predictions refinement module learns the anatomical prior of the breast and pectoral muscle in mammograms, which provides regularization constraints for the target predictions used in the student model training. Therefore, fluctuations in the performance of the teacher model will not have a large impact on the target predictions. Moreover, GAN uses domain-independent semantic features as input, and its main reliance on the teacher model is the high-confidence predictions, which are usually easy to learn. Thus, the low-confidence prediction refinement module can provide regularization constraints for target predictions that do not vary by the data center.

GAN was used in our method as the model for low-confidence prediction refinement, and the loss function of the generator includes a consistency loss and adversarial loss, which can be considered the fidelity and regularity terms, respectively. The consistency loss constrained the generator’s predictions to be consistent with the high-confidence teacher predictions, while the adversarial loss constrained the generator’s predictions to conform to the shape prior given by the annotation. Since GAN’s adversarial loss can use unpaired data, each prediction of the generator is subject to regularization from the entire dataset, preventing the model from directly memorizing the annotations corresponding to the inputs.

Our method draws on doctors’ experience, takes the confident part of the pectoral muscle prediction and shape of the breast as input, and uses a generative adversarial network (GAN) to learn to use this information to refine the low-confidence predictions of the pectoral muscle. Since the dataset distribution of different hospital centers is very different, our method can effectively alleviate this problem and improve the generalization ability of the model. Compared with the baseline method, our method can better mine the latent feature structure in the low-confidence prediction data, thereby improving the segmentation ability of the model. We draw on doctors’ experience in delineation by learning the prior knowledge obtained from annotation and refining the low-confidence predictions. This allows our method to alleviate the confirmation bias problem while providing access to domain-relevant information. The experimental results demonstrate that the proposed method improves the performance of the model on the test set.

The low-confidence prediction refinement module uses the breast segmentation results as an input feature because both the breast border and pectoral muscle are important localization benchmarks and can be used as shape constraints for each other. In addition, coarse segmentation of the breast is usually a relatively easy task compared to segmentation of the pectoral muscle. This provides an idea for other medical image segmentation tasks, where additional regularization constraints can be provided for the current task using simple tasks related to the current task.

## 5. Conclusions

In this study, we proposed an uncertainty-aware semi-supervised segmentation method that incorporates a breast anatomical prior for pectoral muscle segmentation in mammograms. The method uses uncertainty to measure the confidence of predictions and refines the low-confidence predictions by learning task-relevant prior knowledge through a GAN. This improves the quality of the target predictions while allowing the model to learn domain-relevant features in the dataset more efficiently, thereby improving the generalization ability of the model. The experimental results show that compared with the baseline method, the proposed method has an average increase of 1.76 in the DICE index, an average decrease of 3.21 in the IoU index, and an average decrease of 5.48 in the HD index. The experimental results show that our method improves the model performance in pectoral muscle segmentation, reduces unacceptable segmentation results, and provides good generalizability.

The amount of data used in the experiment is limited, and whether the model can be extended to clinical application needs further experimental exploration. A limitation of this study is the lack of testing on a public dataset. However, publicly available datasets generally lack a gold standard for pectoral muscle delineation as well as detailed information on scan data, which is critical for model training. Another limitation of our study is that we did not quantify the low-confidence predictions. The results of low-confidence prediction were all optimized in the low-confidence prediction refinement module. No distinction was made between the confidence levels of the data predictions, so the model may miss information about the features predicted with high confidence. Therefore, distinguishing low-confidence prediction results is still a problem worthy of further study. At present, this study is only implemented based on 635 data from three hospitals. In the future, we plan to generalize it to larger datasets and to more datasets from different hospitals. At the same time, the low-confidence prediction was further quantified, and the method of using low-confidence prediction to enhance the learning ability of the model was explored. Low-confidence prediction is not only a limitation of current models, but also a clinical problem that doctors need to solve urgently. In the future, we will continue to explore new ways to leverage low-confidence prediction data.

## Figures and Tables

**Table 1:** Specifications of the dataset used in our study.

Data Center	Number	Size	Vendor
DC1 ^*a*^	185	2800×2082	SIEMENS
DC2 ^*b*^	236	2812×2082	PLANMED NUANCE
DC3 ^*c*^	184	2457×1890	HOLOGIC

^*a*^ The First Affiliated Hospital of Sun Yat-sen University; ^*b*^ The Sun Yat-sen Memorial Hospital of Sun Yat-sen University; ^*c*^ The NanFang Hospital.

**Table 2:** Experimental settings.

Experiments Group	Trainng Set	Validation Set	Test Set
Exp1	DC1	DC2	DC3
Exp2	DC2	DC3	DC1
Exp3	DC3	DC1	DC2

**Table 3:** Experimental results.

Method Measure	Exp1			Exp2			Exp3			Overall OUSR ↓
	**DICE (↑)**	**IoU (↑)**	**HD (↓)**	**DICE**	**IoU**	**HD**	**DICE**	**IoU**	**HD**	
Supervised	95.45	91.30	16.43	94.35	89.30	18.75	93.54	87.86	21.92	55/635
MT	95.36	91.13	15.48	94.19	89.02	19.24	95.95	92.22	14.54	45/635
Ours	**96.16**	**92.60**	**13.39**	**95.86**	**92.05**	**14.62**	**96.61**	**93.44**	**12.66**	**23/635**

## Data Availability

Data cannot be shared publicly due to privacy protection of the participants and ethical restrictions. For researchers interested in the data, requests can be made to the corresponding authors luyao23@mail.sysu.edu.cn. Researchers with questions about the model code covered in the article may also make a request to the corresponding author luyao23@mail.sysu.edu.cn.

## References

[1] Kwok S., Chandrasekhar R., Attikiouzel Y., Rickard M. (2004). Automatic Pectoral Muscle Segmentation on Mediolateral Oblique View Mammograms. IEEE Trans. Med. Imaging.

[2] Brandt S.S., Karemore G., Karssemeijer N., Nielsen M. (2011). An Anatomically Oriented Breast Coordinate System for Mammogram Analysis. IEEE Trans. Med. Imaging.

[3] Javier Perez-Benito F., Signol F., Perez-Cortes J.C., Fuster-Baggetto A., Pollan M., Perez-Gomez B., Salas-Trejo D., Casals M., Martinez I., LLobet R. (2020). A Deep Learning System to Obtain the Optimal Parameters for a Threshold-Based Breast and Dense Tissue Segmentation. Comput. Methods Programs Biomed..

[4] Maghsoudi O.H., Gastounioti A., Scott C., Pantalone L., Wu F.F., Cohen E.A., Winham S., Conant E.F., Vachon C., Kontos D. (2021). Deep-LIBRA: An Artificial-Intelligence Method for Robust Quantification of Breast Density with Independent Validation in Breast Cancer Risk Assessment. Med. Image Anal..

[5] Karssemeijer N. (1998). Automated Classification of Parenchymal Patterns in Mammograms. Phys. Med. Biol..

[6] Shapiro L., Stockman G. (2001). Computer Vision.

[7] Ferrari R., Rangayyan R., Desautels J., Borges R., Frere A. (2004). Automatic Identification of the Pectoral Muscle in Mammograms. IEEE Trans. Med. Imaging.

[8] Ma W.Y., Manjunath B.S. (2000). EdgeFlow: A Technique for Boundary Detection and Image Segmentation. IEEE Trans. Image Process..

[9] Ma F., Bajger M., Slavotinek J.P., Bottema M.J. (2007). Two Graph Theory Based Methods for Identifying the Pectoral Muscle in Mammograms. Pattern Recognit..

[10] Subashini T.S., Ramalingam V., Palanivel S. (2010). Automated Assessment of Breast Tissue Density in Digital Mammograms. Comput. Vis. Image Underst..

[11] Liu C.C., Tsai C.Y., Liu J., Yu C.Y., Yu S.S. (2012). A Pectoral Muscle Segmentation Algorithm for Digital Mammograms Using Otsu Thresholding and Multiple Regression Analysis. Comput. Math. Appl..

[12] Mustra M., Grgic M. (2013). Robust Automatic Breast and Pectoral Muscle Segmentation from Scanned Mammograms. Signal Process..

[13] Kurt B., Nabiyev V.V., Turhan K. (2014). A Novel Automatic Suspicious Mass Regions Identification Using Havrda & Charvat Entropy and Otsu’s N Thresholding. Comput. Methods Programs Biomed..

[14] Mahersia H., Boulehmi H., Hamrouni K. (2016). Development of Intelligent Systems Based on Bayesian Regularization Network and Neuro-Fuzzy Models for Mass Detection in Mammograms: A Comparative Analysis. Comput. Methods Programs Biomed..

[15] Shen R., Yan K., Xiao F., Chang J., Jiang C., Zhou K. (2018). Automatic Pectoral Muscle Region Segmentation in Mammograms Using Genetic Algorithm and Morphological Selection. J. Digit. Imaging.

[16] Moayedi F., Azimifar Z., Boostani R., Katebi S. (2010). Contourlet-Based Mammography Mass Classification Using the SVM Family. Comput. Biol. Med..

[17] Maitra I.K., Nag S., Bandyopadhyay S.K. (2012). Technique for Preprocessing of Digital Mammogram. Comput. Methods Programs Biomed..

[18] Guo Y., Dong M., Yang Z., Gao X., Wang K., Luo C., Ma Y., Zhang J. (2016). A New Method of Detecting Micro-Calcification Clusters in Mammograms Using Contourlet Transform and Non-Linking Simplified PCNN. Comput. Methods Programs Biomed..

[19] Peng W., Mayorga R.V., Hussein E.M.A. (2016). An Automated Confirmatory System for Analysis of Mammograms. Comput. Methods Programs Biomed..

[20] Feudjio C.K., Klein J., Tiedeu A., Colot O. (2013). Automatic Extraction of Pectoral Muscle in the MLO View of Mammograms. Phys. Med. Biol..

[21] Shi P., Zhong J., Rampun A., Wang H. (2018). A Hierarchical Pipeline for Breast Boundary Segmentation and Calcification Detection in Mammograms. Comput. Biol. Med..

[22] Camilus K.S., Govindan V.K., Sathidevi P.S. (2010). Computer-Aided Identification of the Pectoral Muscle in Digitized Mammograms. J. Digit. Imaging.

[23] Zhou C., Wei J., Chan H.P., Paramagul C., Hadjiiski L.M., Sahiner B., Douglas J.A. (2010). Computerized Image Analysis: Texture-field Orientation Method for Pectoral Muscle Identification on MLO-view Mammograms. Med. Phys..

[24] Tzikopoulos S.D., Mavroforakis M.E., Georgiou H.V., Dimitropoulos N., Theodoridis S. (2011). A Fully Automated Scheme for Mammographic Segmentation and Classification Based on Breast Density and Asymmetry. Comput. Methods Programs Biomed..

[25] Keller B.M., Nathan D.L., Wang Y., Zheng Y., Gee J.C., Conant E.F., Kontos D. (2012). Estimation of Breast Percent Density in Raw and Processed Full Field Digital Mammography Images via Adaptive Fuzzy C-Means Clustering and Support Vector Machine Segmentation. Med. Phys..

[26] Chakraborty J., Mukhopadhyay S., Singla V., Khandelwal N., Bhattacharyya P. (2012). Automatic Detection of Pectoral Muscle Using Average Gradient and Shape Based Feature. J. Digit. Imaging.

[27] Bora V.B., Kothari A.G., Keskar A.G. (2016). Robust Automatic Pectoral Muscle Segmentation from Mammograms Using Texture Gradient and Euclidean Distance Regression. J. Digit. Imaging.

[28] Xie W., Li Y., Ma Y. (2016). Breast Mass Classification in Digital Mammography Based on Extreme Learning Machine. Neurocomputing.

[29] UrviOza, Gohel B., Kumar P. (2024). Evaluation of Normalization Algorithms for Breast Mammogram Mass Segmentation. Procedia Comput. Sci..

[30] Ghoushchi S.J., Ranjbarzadeh R., Najafabadi S.A., Osgooei E., Tirkolaee E.B. (2023). An extended approach to the diagnosis of tumour location in breast cancer using deep learning. J. Ambient. Intell. Humaniz. Comput..

[31] Eklund G., Cardenosa G., Parsons W. (1994). Assessing Adequacy of Mammographic Image Quality. Radiology.

[32] Ma X., Wei J., Zhou C., Helvie M.A., Chan H.P., Hadjiiski L.M., Lu Y. (2019). Automated Pectoral Muscle Identification on MLO-view Mammograms: Comparison of Deep Neural Network to Conventional Computer Vision. Med. Phys..

[33] Ronneberger O., Fischer P., Brox T., Navab N., Hornegger J., Wells W.M., Frangi A.F. (2015). U-Net: Convolutional Networks for Biomedical Image Segmentation. Proceedings of the Medical Image Computing and Computer-Assisted Intervention—(MICCAI) 2015, 18th International Conference.

[34] Rampun A., Lopez-Linares K., Morrow P.J., Scotney B.W., Wang H., Garcia Ocana I., Maclair G., Zwiggelaar R., Gonzalez Ballester M.A., Macia I. (2019). Breast Pectoral Muscle Segmentation in Mammograms Using a Modified Holistically-Nested Edge Detection Network. Med. Image Anal..

[35] Xie S., Tu Z. (2017). Holistically-Nested Edge Detection. Int. J. Comput. Vis..

[36] Guo Y., Zhao W., Li S., Zhang Y., Lu Y. (2020). Automatic Segmentation of the Pectoral Muscle Based on Boundary Identification and Shape Prediction. Phys. Med. Biol..

[37] Yang X., Song Z., King I., Xu Z. (2021). A Survey on Deep Semi-supervised Learning. arXiv.

[38] Tarvainen A., Valpola H. (2017). Mean Teachers Are Better Role Models: Weight-averaged Consistency Targets Improve Semi-Supervised Deep Learning Results. Proceedings of the Advances in Neural Information Processing Systems.

[39] Yu L., Wang S., Li X., Fu C.W., Heng P.A. (2019). Uncertainty-Aware Self-ensembling Model for Semi-supervised 3D Left Atrium Segmentation. arXiv.

[40] Zhang Y., Liao Q., Jiao R., Zhang J. (2021). Uncertainty-Guided Mutual Consistency Learning for Semi-Supervised Medical Image Segmentation. arXiv.

[41] Lai X., Tian Z., Jiang L., Liu S., Zhao H., Wang L., Jia J. Semi-Supervised Semantic Segmentation with Directional Context-aware Consistency. Proceedings of the 2021 IEEE/CVF Conference on Computer Vision and Pattern Recognition, CVPR.

[42] Long M., Cao Y., Cao Z., Wang J., Jordan M. (2019). Transferable Representation Learning with Deep Adaptation Networks. IEEE Trans. Pattern Anal. Mach. Intell..

[43] Vu T.H., Jain H., Bucher M., Cord M., Perez P. ADVENT: Adversarial Entropy Minimization for Domain Adaptation in Semantic Segmentation. Proceedings of the 2019 IEEE/CVF Conference on Computer Vision and Pattern Recognition (CVPR 2019).

[44] Meng Y., Zhang H., Zhao Y., Yang X., Qian X., Huang X., Zheng Y. Spatial Uncertainty-Aware Semi-Supervised Crowd Counting. Proceedings of the 2021 IEEE/CVF International Conference on Computer Vision (ICCV).

[45] Brock A., Donahue J., Simonyan K. Large Scale GAN Training for High Fidelity Natural Image Synthesis. Proceedings of the International Conference on Learning Representations.

[46] Karras T., Laine S., Aila T. A Style-Based Generator Architecture for Generative Adversarial Networks. Proceedings of the 2019 IEEE/CVF Conference on Computer Vision and Pattern Recognition (CVPR 2019).

[47] Xu A., Wang S., Fan J., Shi X., Chen Q. Dual Attention Based Uncertainty-aware Mean Teacher Model for Semi-supervised Cardiac Image Segmentation. Proceedings of the 2021 IEEE International Conference on Progress in Informatics and Computing (PIC).

[48] Kendall A., Gal Y., Guyon I., Luxburg U.V., Bengio S., Wallach H., Fergus R., Vishwanathan S., Garnett R. (2017). What Uncertainties Do We Need in Bayesian Deep Learning for Computer Vision?. Proceedings of the Advances in Neural Information Processing Systems 30 (NIPS 2017).

[49] Weickert J., Michaelis B., Krell G. (2003). Coherence-Enhancing Shock Filters. Pattern Recognition, Proceedings.

[50] Goodfellow I., Pouget-Abadie J., Mirza M., Xu B., Warde-Farley D., Ozair S., Courville A., Bengio Y. (2014). Generative Adversarial Nets. Proceedings of the Advances in Neural Information Processing Systems.

[51] Arjovsky M., Chintala S., Bottou L. Wasserstein Generative Adversarial Networks. Proceedings of the 34th International Conference on Machine Learning, PMLR.

[52] Gulrajani I., Ahmed F., Arjovsky M., Dumoulin V., Courville A.C. (2017). Improved Training of Wasserstein GANs. Proceedings of the Advances in Neural Information Processing Systems.

[53] Kingma D.P., Ba J. (2017). Adam: A Method for Stochastic Optimization. arxiv.

